# Preparation of Electrochemical Sensors Based on Graphene/Ionic Liquids and the Quantitative Detection and Toxicity Evaluation of Tetracycline

**DOI:** 10.3390/nano15040263

**Published:** 2025-02-10

**Authors:** Meidan Lai, Linzhe Huang, Chengzhi Wang, Rui Zuo, Jun Liu

**Affiliations:** 1 College of Water Sciences, Beijing Normal University, Beijing 100875, China; 202431470009@mail.bnu.edu.cn (M.L.); 202431470021@mail.bnu.edu.cn (L.H.); zr@bnu.edu.cn (R.Z.); 2 Guangdong-Hong Kong Joint Laboratory for Water Security, Engineering Research Center of Ministry of Education of Groundwater Pollution Control and Remediation, Center for Water Research, Advanced Institute of Natural Sciences, Beijing Normal University, Zhuhai 519087, China; 3 Engineering Research Center of Groundwater Pollution Control and Remediation, Ministry of Education, Beijing 100875, China

**Keywords:** electrochemistry, tetracycline, graphene, ionic liquid, cytotoxicity

## Abstract

Tetracycline antibiotics, which are recognized as emerging environmental pollutants, are overused and retained in large quantities in terminal water bodies, seriously endangering the ecological environment and human health. Therefore, establishing a straightforward, rapid, and sensitive method for quantitatively detecting and evaluating the toxicity of tetracyclines is highly important. Compared with traditional detection methods, emerging electrochemical methods have many advantages, such as simplicity and rapidity. In this work, an electrochemical sensor—a graphene ionic liquid composite glass carbon electrode (Gr/IL/GCE) with excellent catalytic properties for both tetracycline and cellular purine bases—was prepared by modifying a glassy carbon electrode with graphene and an ionic liquid for the quantitative detection of tetracycline and evaluation of its toxicity to cells. Graphene and the ionic liquid were uniformly distributed on the surface of the electrode and increased the electrically active surface area. The linear range of detection of tetracycline by a Gr/IL/GCE was 10–500 μM, with a detection limit of up to 2.06 μM. The Gr/IL/GCE demonstrated remarkable electrocatalytic efficacy against purine bases within human hepatocellular carcinomas (HepG2) cells. To evaluate the cytotoxicity of tetracycline, the median inhibition concentration (IC_50_) was determined, which was 243.82 μM.

## 1. Introduction

In recent years, the issue of antibiotic residues in the environment has emerged as a significant global public health concern, largely due to the increased antibiotic application frequency, dosages, and emissions. Tetracycline (TC) is recognized as one of the broadly utilized antibiotics around the world [1]. It is employed in the treatment of human and animal diseases, as well as in agricultural feed additives, owing to its broad antimicrobial spectrum and potent therapeutic effects. Statistical data indicate that the global utilization of TC in food animals reached 33,305 tons in 2020 [2]. However, only a minor proportion of ingested tetracycline undergoes metabolic or absorptive processes within the body, while a considerable portion (exceeding 70%) of the TC is excreted from the body in active drug form through urine and feces, subsequently entering the soil and water environment [3]. Tetracycline is chemically stable and resistant to oxidation in the environment, with low volatility and consequently low degradability [4]. Upon entering the environment, tetracycline may persistently disrupt the microbial ecological structure, thereby negatively affecting the physiological functions, reproduction, and development of aquatic organisms. Furthermore, TC residues exert selective pressure on the microbial community, leading to the accumulation and dissemination of antibiotic resistance genes (ARGs) in microorganisms and posing a significant threat to humans through the food chain. Consequently, there is a pressing need to develop effective monitoring strategies and assessment methods for TC residues to ascertain their biotoxicity.

The recent rapid development of electrochemical technology has the potential to overcome the limitations of traditional quantitative detection methods for tetracycline. These include the need for bulky instrumentation for high-performance liquid chromatography [5], poor stability of enzyme-linked immunoassays [6], and suboptimal accuracy and reproducibility of biological detection methods [7]. The broad applicability of electrochemical technology in quantitative detection research makes it an attractive option for further development [8]. Electrochemical detection methods are capable of calculating the relevant information on the desired test object through the signal generated by the test object on an electrode [9]. To establish a simple and sensitive electrochemical detection method for tetracycline, the preparation of composite-modified electrodes of nanomaterials with catalytic activity and electron transfer capability remains a significant area of interest [10].

Toxicological experiments are a fundamental methodology for assessing the toxicity of pollutants in water bodies. As the smallest unit with characteristics of life, cells play an indispensable role in gaining insights into complex biological structures and functions, as well as in toxicity evaluation [11]. The utilization of cell lines for toxicity testing can address numerous limitations associated with in vivo experiments. Cellular electrochemical assays enable the assessment of cellular activity based on the ability of electrodes to recognize specific active ingredients [12]. Among these methods, emerging electrochemical methods based on cellular nucleotide metabolism provide novel means for the electrochemical detection of pollutant toxicity. The fundamental premise is that when the sensing element cells are exposed to varying concentrations of pollutants, the electrochemical signal intensity is influenced by discrepancies in the extent of inhibition of the cells [13]. Nucleotide metabolites such as guanine and xanthine have been identified as sources of cellular electrochemical signals [14]. Consequently, the development of composite-modified electrodes with superior electrocatalytic capabilities through the utilization of innovative nanomaterials represents a prominent area of research.

Carbon nanomaterials represent a central component of contemporary research in the field of new materials. Among the numerous carbon nanomaterials that have been identified to date, graphene is considered one of the most representative examples [15]. Owing to its excellent and stable optical, mechanical, and electrical properties, as well as its unique atom-thick two-dimensional structure and excellent performance, graphene occupies an important position in the synthesis of new materials, the modification of electrodes and electrochemical sensors for electrocatalysis, and energy conversion and storage [16,17]. Ionic liquids (ILs) are a class of compounds consisting exclusively of ions with melting points below 100 degrees Celsius. The first IL (ammonium nitroxide) was reported by Paul Walden in 1914, and ILs have gradually gained attention as novel modifying materials in recent years [18]. ILs possess various advantageous properties, including excellent biocompatibility and conductivity [19]. Additionally, they exhibit sufficiently wide potential windows for electrochemical detection, which makes them ideal for use as green solvents and dispersants in electrochemistry [20]. In accordance with the kinetic law of solution reactions, viscous media result in diffusion limitations [21]. In addition, in ionic ILs, the lower the degree of cation planarity is, the lower the conductivity [22]. Graphene and ionic liquids can be connected through covalent bonds to enhance sorting and tribological properties [23]. It has been found that the combination of Gr and IL can produce composite films and improve the practical performance of electrochemical sensors [24].

Accordingly, in this study, imidazolium salt ILs were selected for combination and milling with Gr to prepare nanocomposite-modified electrodes with positive catalytic activity against tetracycline and purine, and the electrodes were characterized via various methods. By studying the electrochemical behavior of tetracycline at the electrode, the relationship between the tetracycline concentration and oxidation peak current was analyzed, and the linear detection range and detection limit were determined. Human hepatocellular carcinomas (HepG2) cells were used as models, and the excellent electrocatalytic activity of the composite-modified electrode for purines released after cell lysis due to toxicity was employed to evaluate the toxicity of tetracycline to HepG2 cells. This study simultaneously realizes the quantitative detection and toxicity evaluation of tetracycline and provides new research ideas for the comprehensive study of tetracycline.

## 2. Materials and Methods

### 2.1. Reagents and Materials

HepG2 cells, along with fetal bovine serum (FBS), Dulbecco’s modified Eagle’s medium (DMEM), and phosphate-buffered saline (PBS), were all obtained from Corning Corporation in the United States (Corning, NY, USA). Graphene was acquired from Nanjing XFNANO Materials Tech Co., Ltd. (Nanjing, China). Imidazole salts, which were selected as the ionic liquids, were purchased along with xanthine, guanine, and tetracycline from J&K Scientific (Beijing, China). Hydrogen chloride (HCl) was purchased from DM Chemical Reagent Factory (Tianjin, China). Potassium ferricyanide (K_3_[Fe(CN)_6_]) was purchased from Sinopharm Chemical Reagent Co., Ltd. (Shanghai, China).

### 2.2. Apparatus

Electrochemical experiments were conducted in a three-electrode system purchased from CH Instruments (Shanghai, China) comprising a graphene and ionic liquid modified glassy carbon electrode (Gr/IL/GCE) as the working electrode, a saturated calomel electrode as the reference electrode, and a platinum wire as the counter electrode. All the electrodes were purchased from Gaossunion (Wuhan, China). The surface morphologies of the materials were examined via a JEOL 6340F scanning electron microscope (SEM) from JEOL Ltd. (Tokyo, Japan). The element compositions were characterized via X-ray diffraction. The samples were subjected to analysis via a Rigaku D/MAX-2500 diffractometer, a Japanese instrument (Rigaku Corporation, Tokyo, Japan), with the results presented in the form of diffractograms, and the samples were also analyzed physically, with measurements of their phase, crystallinity, and lattice parameters taken. Raman scattering spectroscopy was performed via a Jobin-Yvon-HR-800 spectrometer (HORIBA, Ltd., Kyoto, Japan). A BDS-400 inverted biomicroscope (Chongqing Optical Instruments Co., Ltd., Chongqing, China) was used to observe the morphology and number of HepG2 cells.

### 2.3. Preparation of the Nanocomposite-Modified Electrode

The polished glassy carbon electrode (GCE, diameter = 3 mm) was rinsed with ultrapure water and subsequently dried. Two milligrams of single-layer graphene was added to 2 mL of ultrapure water, and the solution was thoroughly mixed and sonicated for 30 min to obtain a graphene dispersion. A 5 μL aliquot of the suspension was applied to the GCE and irradiated with an infrared lamp to evaporate the solvent. The sample was then allowed to air dry at room temperature to yield the Gr/GCE.

**Figure 1:**
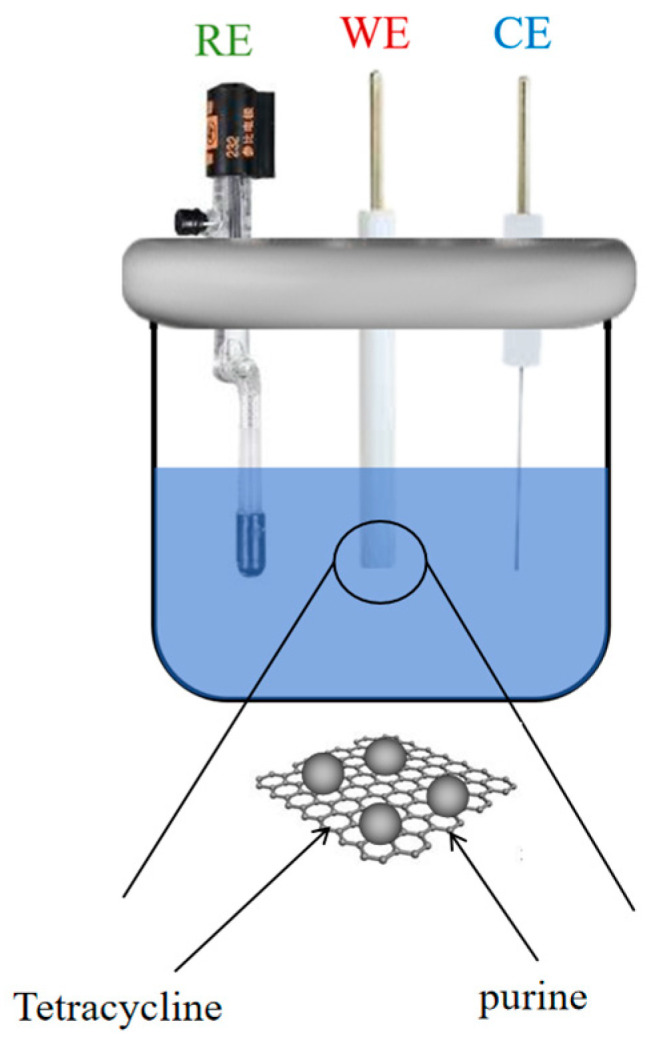
Sensor structure diagram.

A black colloid (Gr/IL) was prepared by placing 7 mg of monolayer graphene and 100 μL of ionic liquid in an onyx mortar and grinding the mixture uniformly for 30 min. The Gr/IL was uniformly deposited onto the surface of the GCE, resulting in the formation of the Gr/IL/GCE. The assembled electrode chamber is shown in Figure 1 (RE is reference electrode, WE is working electrode, CE is counter electrode). The electrodes were subjected to a regeneration process by immersion in PBS with 5 cycles of cyclic voltammetry scanning over a voltage range of 0 to +0.8 V following each use.

### 2.4. Cytotoxicity Assay

The HepG2 cell line was grown in fully supplemented DMEM at 37 °C in 5% CO_2_ and subcultured every 2 days. After cell culture, complete culture medium containing 50 μM, 150 μM, 300 μM, 500 μM, or 800 μM tetracycline was added for an additional 30 h, followed by washing with PBS (pH = 7.4). The requisite quantity of PBS (pH = 7.4) was subsequently introduced, a sealing film was affixed, and the apparatus was placed in a thermostatic water bath box and heated at 50 °C for 30 min to obtain the HepG2 cell lysate.

### 2.5. Electrochemical Detection Method

The three electrodes were individually immersed in tetracycline or cell lysate, and a 0 V potential was applied for 360 s to facilitate the formation of a uniform tetracycline or cell indicator analysis layer on the electrode surface. Subsequently, cyclic voltammetry was conducted to record the oxidation peak current. Following the completion of the experiment, the electrodes were placed in PBS for regeneration.

### 2.6. Statistical Analysis

Three sets of parallel tests were conducted, and the mean and standard deviation were calculated. The resulting data were analyzed and processed via Origin 2021 (Originlab, Northampton, MA, USA) and GraphPad Prism 9.5 (GraphPad Software, San Diego, CA, USA) software.

## 3. Results and Discussion

### 3.1. Characterization of the Gr/IL/GCE

**Figure 2:**
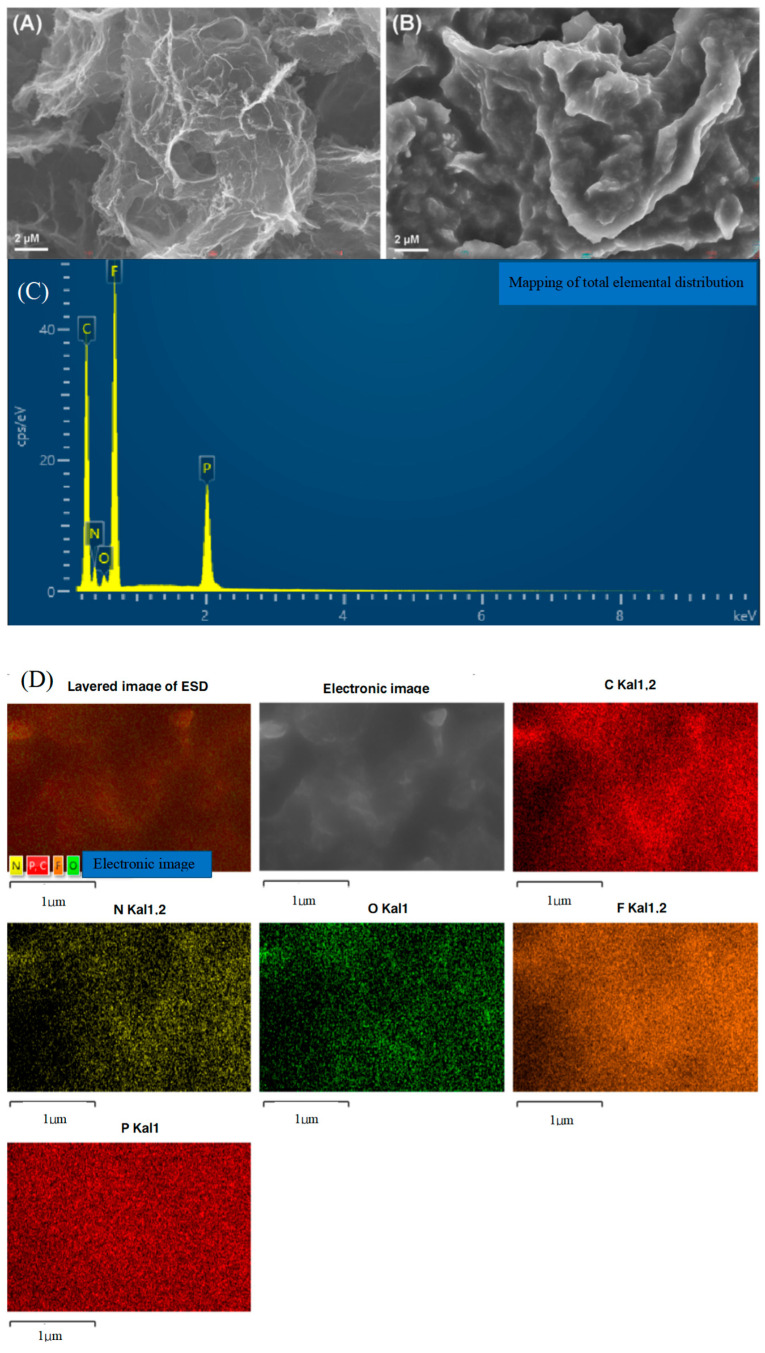
(**A**,**B**) SEM images of the Gr/IL/GCE, (**C**) typical EDS spectrum of the Gr/IL/GCE, and (**D**) layered mapping image of each element.

Scanning electron microscopy (SEM) was employed to elucidate the surface morphology of the Gr and Gr/IL, and the findings are presented in Figure 2A,B. Figure 2A shows that initially, Gr had a conventional sheet structure of monolayer graphene. Figure 2B shows that after the mixed milling treatment of the IL and Gr, the Gr was enveloped by the IL. The IL was also situated between the monolayer graphene. The inherent viscosity of the IL enabled the formation of robust interconnections between the Gr particles. The incorporation of the IL not only improved the dispersion of graphene sheets but also endowed the graphene with a positive charge, which promoted the formation of multilayer films [25].

EDS was used to characterize the elemental composition of the composite material, and the results are shown in Figure 2C. The figure shows that the composite material consisted mainly of C, N, O, F, and P. The elements C and O originated from the Gr, and the elements N, F, and P are attributed to the IL. Figure 2D shows that the elements from the Gr and IL were uniformly distributed on the surface of the material, which suggests that they were sufficiently mixed. The findings above validate the successful synthesis of the Gr/IL binary composite nanomaterials.

**Figure 3:**
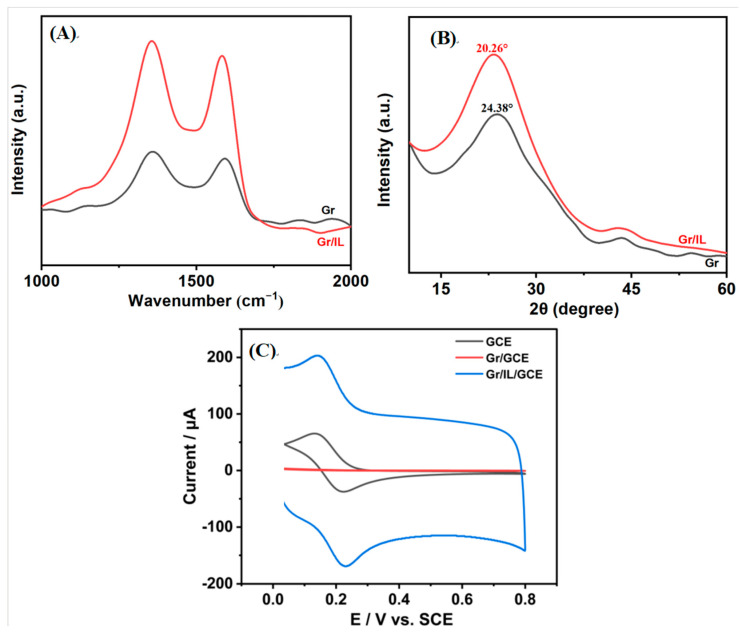
(**A**) Raman spectra of Gr and Gr/IL; (**B**) XRD patterns of Gr and Gr/IL; (**C**) and CV diagrams of the GCE, Gr/GCE, and Gr/IL/GCE in the K_3_[Fe(CN)_6_] solution.

Raman spectroscopy is employed as a method that is used to characterize the structure of carbon materials. This is achieved by analyzing the vibrational energy level differences of the samples. The technique offers a number of advantages, including the ability to provide fast, easy-to-use, reproducible, and nondestructive qualitative and quantitative analysis. Among these characteristics, the relative intensities of the D and G peaks (ID/IG) can be used to reflect the degree of integrity of the graphite structure of the material [26]. As illustrated in Figure 3A, the ID and IG values for Gr were 9.73 and 11.06, respectively, whereas those for Gr/IL were 26.49 and 31.12, respectively. The calculations indicate that the ID/IG ratio for Gr/IL was 0.85, and the ID/IG ratio for Gr was 0.88, indicating a decrease in the number of lattice defects and an increase in the degree of graphitization.

X-ray diffraction (XRD) is a fundamental structural technique that enables the qualitative and quantitative analysis of the physical phases present in a given sample. X-ray diffraction was employed to analyze Gr and Gr/IL, and the results are presented in Figure 3B.

The diffractograms for pure Gr and Gr-IL lacked the distinctive, intense, and sharp peaks associated with graphite at a 2θ angle of approximately 26° [27]. Instead, a faint peak of minimal intensity was observed at this position, suggesting the lack of well-ordered structures and the exfoliated state of both Gr and Gr-IL. In the XRD spectrum of Gr, a strong diffraction peak was observed at 2θ = 24.38°, and a corresponding characteristic diffraction peak was evident at 2θ = 20.26° following the addition of the IL; this may be attributable to the increased viscosity of the IL, which exerted macroscopic tensile stresses on the Gr after grinding with the IL, resulting in the displacement of the characteristic diffraction peaks to lower angles [28].

Cyclic voltammetry (CV) serves as an effective analytical technique for probing the electrochemical characteristics of substances, deciphering reaction mechanisms, and uncovering the redox behaviors within a system [29]. The electrochemical behavior of different electrodes in K_3_[Fe(CN)_6_] solutions was investigated via cyclic voltammetry, and the results are shown in Figure 3C. Gr/GCE detected two signals located at +0.13 V and +0.22 V, with peak current magnitudes of 65.39 μA and 37.42 μA, respectively, whereas the binary composite nanomodified electrode Gr/IL/GCE detected distinct oxidation peaks and reduction peaks at +0.14 V and +0.23 V, respectively, with significantly larger peak current values of 203.14 μA and 168.95 μA, respectively. The above results indicate that the Gr/IL/GCE has excellent electron transfer performance for potassium ferricyanide, which is mainly attributed to the following factors: first, the π–π interactions between the imidazole ring of bmim-PF6 and the graphene sheets [30] are favorable for the electrochemical reaction; second, the IL helps the dispersion of Gr, and the composite forms a stable colloidal material, which is favorable for electron transfer. The electrically active surface area of the Gr/IL/GCE was calculated via equation (1) to be 1.58 cm^2^, which was 1.9 times that of the GCE.

(1) $$I_{p}=\left(2.99\times 10^{5}\right){nACD}^{\frac{1}{2}}v^{\frac{1}{2}}$$

In this equation, Ip represents the peak current, n represents the number of electrons participating in the redox reaction, A represents the electroactive surface area, C represents the concentration of the K_3_[Fe(CN)_6_] solution, D represents the diffusion coefficient (6.30 × 10^6^ cm^2^·s^−1^), and v represents the scanning rate [31].

### 3.2. Quantification of Tetracycline by the Gr/IL/GCE

**Figure 4:**
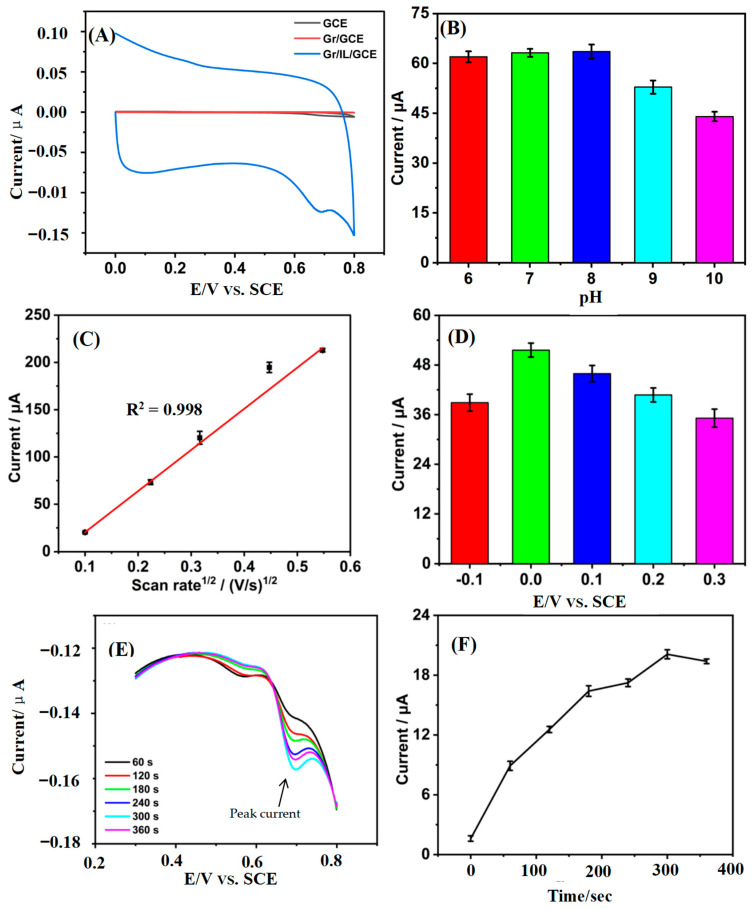
(**A**) Cyclic voltammograms of 200 μM tetracycline on the GCE, Gr/GCE, and Gr/IL/GCE. (**B**) Effect of pH on the oxidation peak current of 200 μM tetracycline on the Gr/IL/GCE. (**C**) Effect of scan speed on the oxidation peak current of 200 μM tetracycline on the Gr/IL/GCE. (**D**) The influence of enrichment capability on the oxidative peak current response of 200 μM tetracycline at the Gr/IL/GCE surface. (**E**) Effect of enrichment time on the CV diagram and (**F**) oxidation peak current of tetracycline on the Gr/IL/GCE.

Tetracycline solutions of different concentrations are used as electrolytes in the quantitative study of tetracycline. The voltammetric behaviors of tetracycline on the GCE, Gr/GCE, and Gr/IL/GCE were investigated (Figure 4A). No discernible oxidation peak was observed for either the GCE or the Gr/GCE. In contrast, the Gr/IL/GCE exhibited a prominent oxidation peak at +0.68 V with a peak current of 200.17 μA, which was markedly superior to those of the GCE and the Gr/GCE. This finding suggests that the Gr and IL composite-modified materials possess better catalytic capabilities.

Figure 4 shows the effects of different pH values (6, 7, 8, 9, and 10) (Figure 4A), different scan rates (0.01, 0.05, 0.1, 0.2, and 0.3 V/S) (Figure 4B), different enrichment potentials (−0.1~3 V) (Figure 4C), and different enrichment times (0 s–360 s) (Figure 4D) on the electrochemical response signals of tetracycline on the Gr/IL/GCE. The enrichment conditions such as potential and enrichment time have certain influence on the subsequent electrochemical behavior [32]. Among the observed phenomena, the oxidation peak current decreased when the enrichment time exceeded 300 s, indicating saturation of the Gr/IL/GCE surface with electroactive substances at 300 s. A direct proportionality exists between the oxidation peak current and the quadratic root of the scanning rate, and the linear equation is I_pa_ (μA) = 435.22 v^1/2^ (mV/s) − 23.14 (R^2^= 0.998). The reaction process of tetracycline on the Gr/IL/GCE was influenced mainly by diffusion. If the scan rate is too high, insufficient utilization of the active substance can easily occur, thus affecting the detection results; therefore, the commonly used scan rate of 0.05 V/s is generally chosen for cyclic voltammetry scanning. The maximum oxidation peak current of tetracycline was detected at pH = 8.0, a cyclic voltammetry scan rate of 0.05 V/s, an enrichment potential of 0 V, and an enrichment time of 300 s. Therefore, these conditions were determined to be the optimal preparation conditions for the quantitative detection of tetracycline.

**Figure 5:**
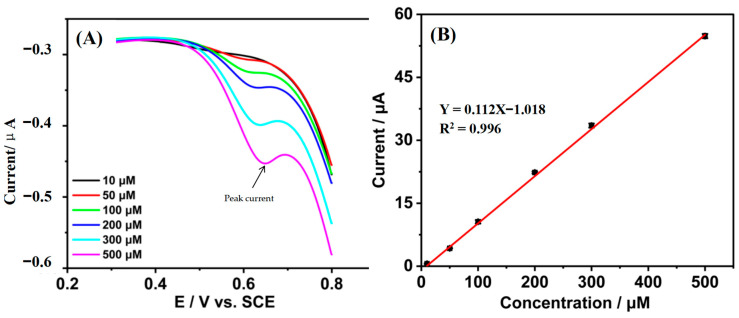
CV plots (**A**) and oxidized peak current values (**B**) for different concentrations of tetracycline on the Gr/IL/GCE.

In the most favorable experimental setting, CV was used to detect the electrochemical response of tetracycline at Gr/IL/GCE at a scanning speed of 0.05 V/s (Figure 5A). The peak current was around 0.68 V for all concentrations of tetracycline. A favorable linear relationship was identified between the oxidation peak current (I_pa_) of tetracycline and its concentration (c) within the range of 10–500 μM (Figure 5B), and the linear equation is I_pa_ (μA) = 0.112 c (μM) − 1.018 (R^2^ = 0.996). According to equation (2), the detection limit is 2.06 μM.

(2) $$\mathrm{Limit}\;\mathrm{of}\;\mathrm{Detection}\;(\mathrm{LOD}):\;LOD=3.3\times \frac{\sigma }{S}$$

where σ is the standard deviation of the response value, and S is the slope of the standard curve.

A comparison of the results of tetracycline detection by the modified electrodes reported in this study with those of the Gr/IL/GCE (Table 1) revealed that the Gr/IL/GCE has a wider linear detection range and a lower detection limit under simpler preparation conditions.

According to previous studies on the residual amount of tetracycline in the environment by various analytical methods, it can be known that the concentration of tetracycline in the environment can reach 2.63 μM [8]. According to the experimental results, Gr/IL/GCE has the potential to be used in real water as a new electrode with lower LOD and larger detection range.

### 3.3. Toxicity of Tetracycline to HepG2 Cells

**Figure 6:**
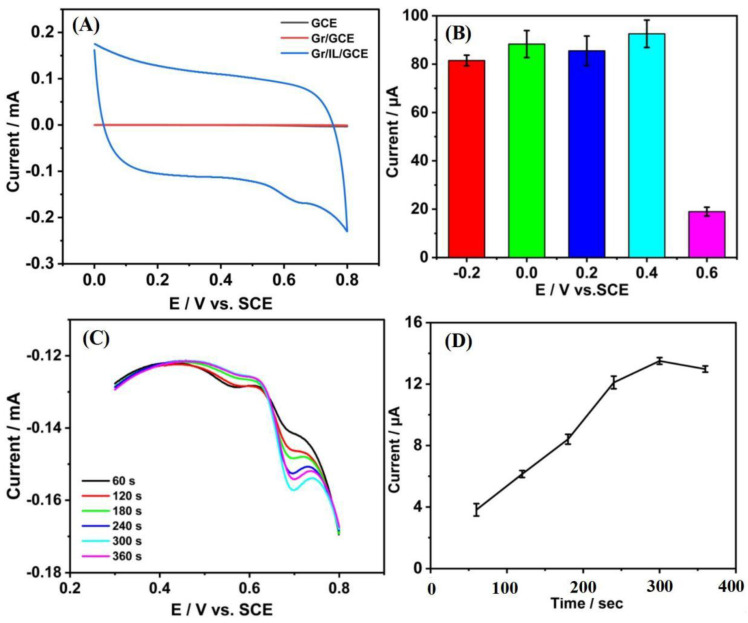
(**A**) Cyclic voltammograms of HepG2 cells on the GCE, Gr/GCE, and Gr/IL/GCE. (**B**) Effect of the concentration potential of the 50 μM purine standard mixture on the oxidation peak current on the Gr/IL/GCE, (**C**) effect of enrichment time on the CV diagram, and (**D**) oxidation peak current of the purine standard mixture on the Gr/IL/GCE.

The electrochemical experiments were carried out by using cell lysates that were lysed by poisoning with different concentrations of tetracycline as an electrolyte. The cyclic voltammetric behavior of the cells on the GCE, Gr/GCE, and Gr/IL/GCE was examined (Figure 6A). The detection of cytotoxicity by electrochemical sensors is mainly carried out through the electrocatalysis of purines, which are not produced until after cell lysis [39,40]. No oxidation peaks of purines were detected on the GCE or the Gr/GCE. However, a more pronounced oxidation peak was observed for the Gr/IL/GCE, indicating that the Gr/IL interface may facilitate the oxidation of purines. A peak was identified for the Gr/IL/GCE at a potential of +0.647 V, with a peak current of 11.43 μA. The Gr/IL composite has been shown to effectively enhance electrocatalytic performance in the detection of cellular electrochemical signals. Additionally, the absence of a reduction peak in the research suggests that the oxidation of electroactive materials on the Gr/IL/GCE is not reversible.

The effects of different enrichment potentials (−0.2 V, 0.2 V, 0 V, +0.2 V, +0.4 V, and +0.6 V) (Figure 6B) and different enrichment times (0–360 s) (Figure 6C,D) on the oxidation peak currents of the tetracycline standards on the Gr/IL/GCE were investigated. The maximum oxidation peak current of the purine standard mixture was observed when 0.4 V was the enrichment potential for cellular electrochemical detection and the enrichment time was 300 s. Therefore, these conditions were determined to be the optimal preparation conditions for the detection of tetracycline cytotoxicity.

**Figure 7:**
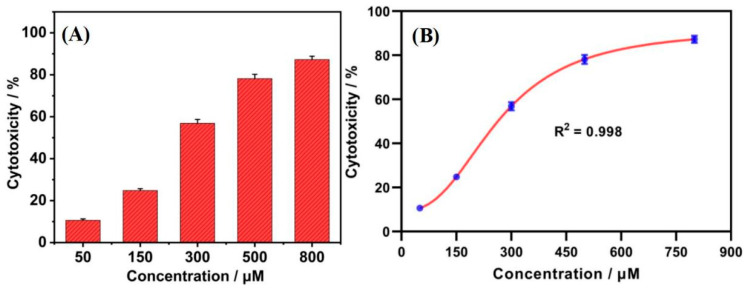
(**A**) Toxicity of different concentrations of tetracycline to HepG2 cells and (**B**) fitting curve of the tetracycline concentration to the toxicity value.

HepG2 cells were stained with different concentrations of tetracycline (50, 150, 300, 500, and 800 μM). The correlation between tetracycline concentration and the peak current of purine oxidation in HepG2 cells was investigated by using a Gr/IL/GCE-based electrochemical method. As shown in Figure 7A, tetracycline had toxic effects on HepG2 cells. The cytotoxicity of tetracycline showed a clear “S-shaped” curve relationship, as shown in Figure 7B. The half inhibitory concentration (IC_50_) value of tetracycline for HepG2 cells after 30 h was 243.82 μM based on Gr/IL/GCE cell electrochemistry.

### 3.4. Repeatability of the Gr/IL/GCE

To evaluate the reproducibility of the Gr/IL/GCE, the relative standard deviation (RSD) of the peak currents was calculated, which was 4.63% for the Gr/IL/GCE in 200 μM tetracycline and 4.79% for the Gr/IL/GCE in 100 μM purine standard over five consecutive cyclic voltammetry scans, with the same method used for phase preparation in both cases. The RSD of the oxidized peak current was 3.02% for the same 200 μM tetracycline when five Gr/IL/GCEs were prepared in the same phase via the same method, and the RSD of the oxidized peak current was 4.36% when the 100 μM purine standard was detected. The above results confirmed that the Gr/IL/GCE has good reproducibility.

## 4. Conclusions

Gr/IL/GCEs with wide potential windows, high electrocatalytic activity, and good reproducibility can be prepared via a simple scraping-coating method and electrochemical methods, and the characterization results show that Gr can be successfully compounded with an IL and that the IL can effectively disperse Gr, improve the electron transfer performance, and increase the electroactive surface area.

Gr/IL/GCEs have good electrocatalytic performance for tetracycline and can be used for the sensitive detection of tetracycline under optimal detection conditions (enrichment time: 300 s; enrichment potential: 0 V; and pH = 8.0), with a linear range of 10–500 μM and a limit of detection of up to 2.06 μM. Thus, they provide a simple, rapid, and technological method for the quantitative detection of tetracycline in water environments.

The prepared Gr/IL/GCE showed good catalytic performance against electroactive substances in HepG2 cells, and a distinct oxidation peak was measured at +0.68 V. The toxicity of tetracycline to HepG2 cells was investigated under optimal enrichment conditions (enrichment time: 300 s and enrichment potential: +0.4 V), and a significant dose–effect relationship was identified. The 30 h IC_50_ of tetracycline was 243.82 μM. Thus, Gr/IL/GCEs provide a simple and rapid technical means for studying the toxicity of tetracycline in the environment.

## Figures and Tables

**Table 1:** Comparison of the detection limits and ranges of the Gr/IL/GCE and other tetracycline detection sensors.

Modified Electrode	Linear Detection Range (μM)	Detection Limit (μM)	Sensitivity (mA.L/mol)
MIOPPy-AuNP/SPCE [33]	1~20	0.65	-
Ag NCs [34]	1.12~230	0.47	-
PtNPs/C/GCE [35]	9.99~44.01	4.28	45.4
CDs-Eu@paper [36]	0.1~100	0.03	-
clay-CPE [37]	0.1~45	0.16	1100
Fluorescent assay [38]	10~350	1.70	-
Gr/IL/GCE	10~500	2.06	112

## Data Availability

The raw data supporting the conclusions of this article will be made available by the authors on request.
